# Antibacterial and deodorizing effects of cold atmospheric plasma-applied electronic deodorant

**DOI:** 10.1038/s41598-024-53285-9

**Published:** 2024-02-06

**Authors:** Junsoo Bok, Jongbong Choi, Solpa Lee, Tae Ho Lim, Yongwoo Jang

**Affiliations:** 1 Department of Medical and Digital Engineering, College of Engineering, Hanyang University, Seoul, 04736 South Korea; 2 Department of Emergency Medicine, Hanyang University Hospital, Seoul, 04763 South Korea; 3 Department of Pharmacology, College of Medicine, Hanyang University, Seoul, 04736 South Korea; 4 Department of Medical and Digital Engineering, College of Medicine, Hanyang University, Seoul, 04763 South Korea

**Keywords:** Quality of life, Biomedical engineering

## Abstract

Axillary odor is a malodor produced by bacterial metabolism near the apocrine glands, which often causes discomfort in an individual's daily life and social interactions. A deodorant is a personal care product designed to alleviate or mask body odor. Currently, most deodorants contain antimicrobial chemicals and fragrances for odor management; however, direct application to the underarm skin can result in irritation or sensitivity. Therefore, there is a growing interest in technologies that enable disinfection and odor control without the antiperspirants or perfumes. The cold atmospheric plasma temporally generates reactive radicals that can eliminate bacteria and surrounding odors. In this study, cultured *Staphylococcus hominis* and *Corynebacterium xerosis*, the causative bacteria of axillary bromhidrosis, were killed after 90% plasma exposure for 3 min. Moreover, the electronic nose system indicated a significant reduction of approximately 51% in 3-hydroxy-3-methylhexanoic acid and approximately 34% in 3-methyl-3-sulfanylhexan-1-ol, the primary components of axillary odor, following a 5-min plasma exposure. These results support the dual function of our deodorant in eliminating bacteria and axillary odors without the chemical agents. Therefore, cold atmospheric plasma-applied deodorant devices have great potential for the treatment and management of axillary odors as a non-contact approach without chemical use in daily life.

## Introduction

Axillary bromhidrosis, arising from excessive secretion by the apocrine glands, leads to the generation of an axillary odor when this secretion is metabolized by gram-positive bacteria such as *Staphylococcus hominis* (*S. hominis*) and *Corynebacterium xerosis* (*C. xerosis*). This malodor causes discomfort in daily life and social interactions. In some cases, surgery is considered to remove the apocrine glands. However, this surgical procedure is primarily recommended for severe cases owing to the associated risks, such as skin necrosis, hematoma, and scarring. Individuals with milder cases often turn to products such as antiperspirants or perfumes as alternatives to surgery. Although this is a straightforward approach, it is neither a fundamental nor a highly effective solution. Therefore, it is imperative to develop effective solutions to enhance the personal, social, and medical well-being of affected individuals. Thus, it is essential to develop treatments with minimal side effects that are suitable for daily use and are capable of addressing the root causes of axillary odor.

A deodorant is a personal care product designed to help reduce or mask body odors. Currently, most deodorants used by patients with axillary bromhidrosis contain antimicrobial chemicals, such as triclosan and aluminum salts, for antimicrobial effect, along with fragrances for odor management. These antimicrobial agents can cause irritation or sensitivity when applied directly to underarm skin^1^. Notably, their exposure is also known to increase the risk of Alzheimer's disease, breast cancer, and prostate cancer^1–5^. Therefore, there is a growing interest in technologies that can be disinfected and deodorized without relying on antimicrobial agents or products such as antiperspirants or perfumes.

Cold atmospheric plasma is a non-thermal technology that ionizes neutral gases at room temperature and atmospheric pressure using a specific electrode arrangement and discharge conditions^6,7^. Plasma discharge temporarily generates neutral particles, ions, radicals, electrons, and ultraviolet (UV) radiation^8,9^. Due to its high nitrogen-to-oxygen ratio at atmospheric pressure, it generates reactive oxygen species (ROS) and reactive nitrogen species (RNS). These active species disrupt the outer membrane of the bacteria, resulting in sterilization^8,10^. Cold plasma technology is frequently employed for food sterilization because of its non-contact and temporary generation of ROS and RNS^11^. Other reactive species, including ozone, transform odorous volatile organic compounds (VOCs) into alternative degradation products, resulting in efficient VOC removal. This effect has been extensively studied in the fields of waste gas purification and deodorization^12–15^. Therefore, non-thermal plasma technology presents a remarkable potential to address both bacterial sterilization and odor elimination through a non-contact approach without the need for chemical agents^8,9,12–17^. In cases of axillary bromhidrosis where both functions are required, cold atmospheric plasma technology can be applied to deodorant devices for disease treatment and management.

In this study, we developed a novel deodorant device that incorporates cold atmospheric plasma technology. This device was designed to enable individuals to easily apply the main components of axillary bromhidrosis to their armpits. In the functional experiments, we validated the sterilization efficacy of the device employing *S. hominis* and *C. xerosis*, the microorganisms responsible for the axillary odor. Moreover, we assessed the deodorizing capability after plasma exposure using an electronic nose system. These results strongly support the dual functions of bacterial sterilization and axillary odor elimination achieved by the proposed device without the use of chemicals. Therefore, cold atmospheric plasma-applied deodorant devices have great potential for the treatment and management of axillary odors.

## Results and discussion

### Cold plasma-applied electronic deodorant

**Figure 1:**
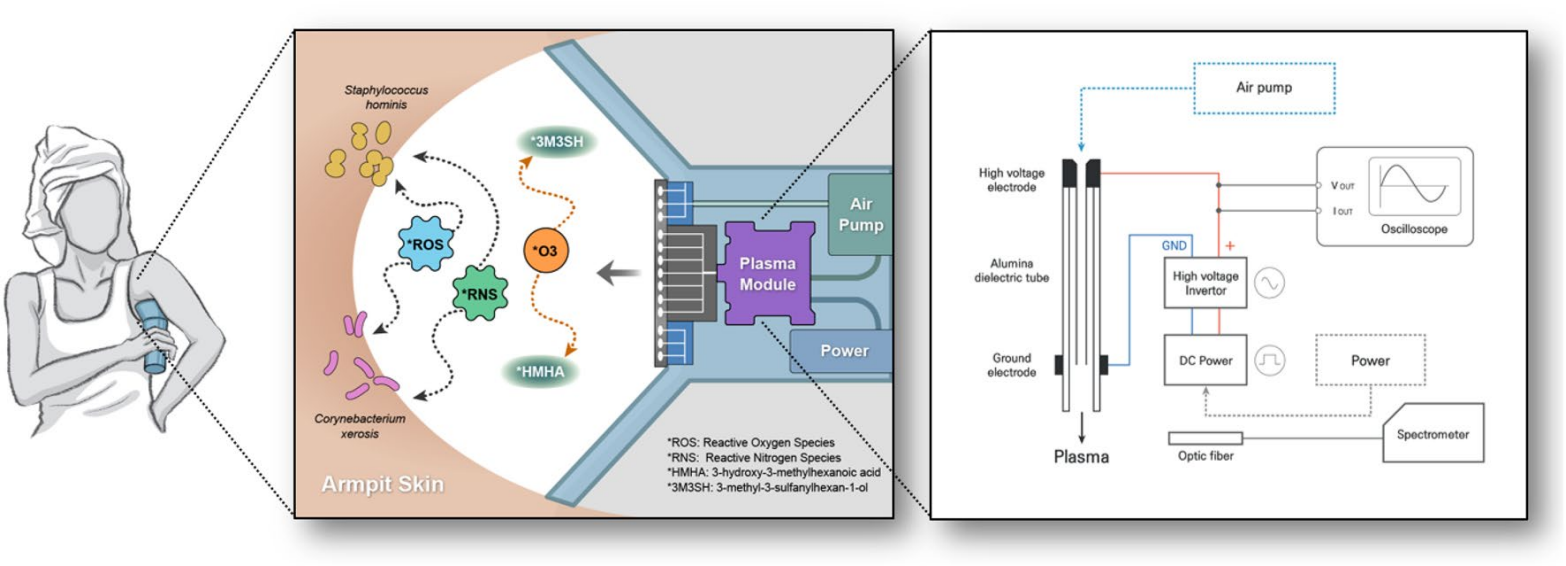
The schematic diagram of cold atmospheric pressure plasma-applied electronic deodorant. The schematic diagram presents the cold atmospheric-pressure plasma-applied electronic deodorant, depicting the mechanism of action involved in sterilization and deodorization.

Figure 1 shows a schematic diagram and action mechanism of the electronic deodorant. The deodorant comprises a plasma module, an air pump, and a power supply. The plasma module included a hollow electrode, an alumina dielectric with a thickness of 1 mm, and an external ground electrode with a width of 5.5 mm. An air pump was incorporated to circulate the reactive radicals generated during plasma discharge, maintaining a flow rate of 2.7 standard liters per minute. The DC input source (12 V, 0.417 A) was converted to AC power using a push–pull type inverter. In ambient air, plasma discharge temporarily generates neutral particles, ions, radicals, electrons, and ultraviolet (UV) radiation^8,9^. Owing to its high nitrogen-to-oxygen ratio at atmospheric pressure, it primarily induces the generation of reactive oxygen species (ROS) and reactive nitrogen species (RNS)^18^. These active species disrupt the outer membrane of the bacteria, resulting in sterilization^8,10^. Of the generated radicals, ozone can react with the odor molecules by transferring the extra oxygen atom of the ozone molecule to the odor molecule, thereby functioning as a deodorizing agent.

### Physical properties of plasma deodorant

**Figure 2:**
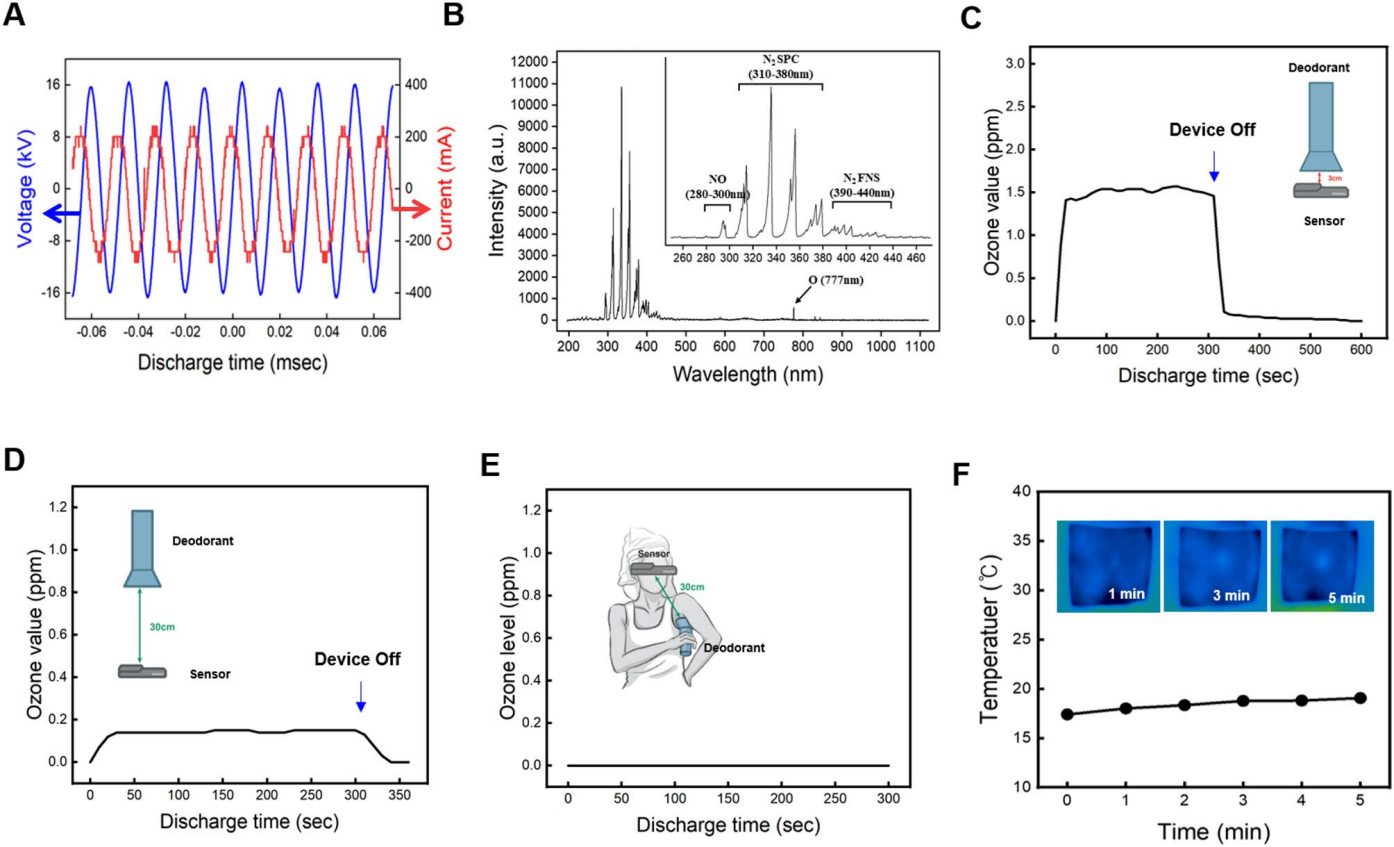
Physical properties of plasma deodorant. (**A**) Current and voltage waveforms during discharge; (**B**) Optical Emission Spectra (OES) of the plasma; (**C**–**E**) ozone generation at different distances (3 cm in open state, 30 cm in open state, and 30 cm in closed state); (**F**) thermal camera images depicting temperature changes in porcine skin over time during discharge.

First, we assessed the physical properties of cold atmospheric plasma in our deodorant. Figure 2A shows the typical current and voltage discharge waveforms generated during plasma discharge in the plasma generation process. The peak discharge current reaches a maximum of 241 mA, and this discharge occurs under a peak voltage of 16.4 kV at a frequency of 62 kHz. Among the components generated by plasma, ROS and RNS play crucial roles in killing bacteria. Therefore, these reactive species were verified using optical emission spectroscopy (OES). As shown in Fig. 2B, the plasma device displayed prominent peaks in the N2 second positive system (SPS) lines, covering the range of 310–380 nm (specifically at 316 nm, 337 nm, 357 nm, and 376 nm). In addition, fainter peaks were observed in the N2 first-positive system (FNS) lines, which extended from 390 to 440 nm (specifically at 399 nm, 405 nm, and 427 nm). We also detected a faint line in the wavelength range of 280–300 nm, which corresponds to NO^19^, and identified an atomic oxygen line at a wavelength of 777 nm^7^. ROS and RNS peaks were observed in the ranges of 250–425 nm according to the Ref.^19^. These results indicated the presence of nitrogen, atomic oxygen, hydroxyl radicals, ROS, and RNS in the generated plasma^8,10^.

Because of the deodorizing capability of the generated ozone, we measured the ozone concentration during plasma discharge at a distance of 3 cm. Plasma discharge elevated the ozone level to 1.5 ppm, which disappeared completely when the plasma was turned off (Fig. 2C). It is well known that inhalation of ozone can be hazardous to the respiratory tract. Therefore, international safety standards for occupational exposure to ozone during working hours include the time-weighted average (TWA) and short-term exposure limit (STEL). The permissible ozone concentration limits were established at TWA 0.05 ppm for 8 h and STEL 0.20 ppm for a 15 min interval. When the distance from the armpit to the nose was approximately 30 cm, the ozone concentration measured 0.16 ppm when measured in a straight line at a distance of 30 cm (Fig. 2D) and 0 ppm when measured diagonally at the same distance (Fig. 2E). These values are notably lower than international safety standards.

Figure 2F shows the temperature changes following plasma discharge on porcine skin. Before the plasma discharge, the temperature of the pig skin was 17.40 °C, which is equivalent to room temperature. After 5 min of discharge, the temperature of the porcine skin increased to 19.06 °C, resulting in a total increase of 1.66 °C (Fig. 2F). This temperature change is notably lower than that of existing plasma devices in dermatology, which typically aim to generate heat below 40 °C^20–23^. This lower thermal effect is mainly attributed to the cold atmospheric plasma. In addition, the air circulation system of our plasma device helps maintain a consistent temperature during plasma discharge.

### Antibacterial effect of plasma deodorant

**Figure 3:**
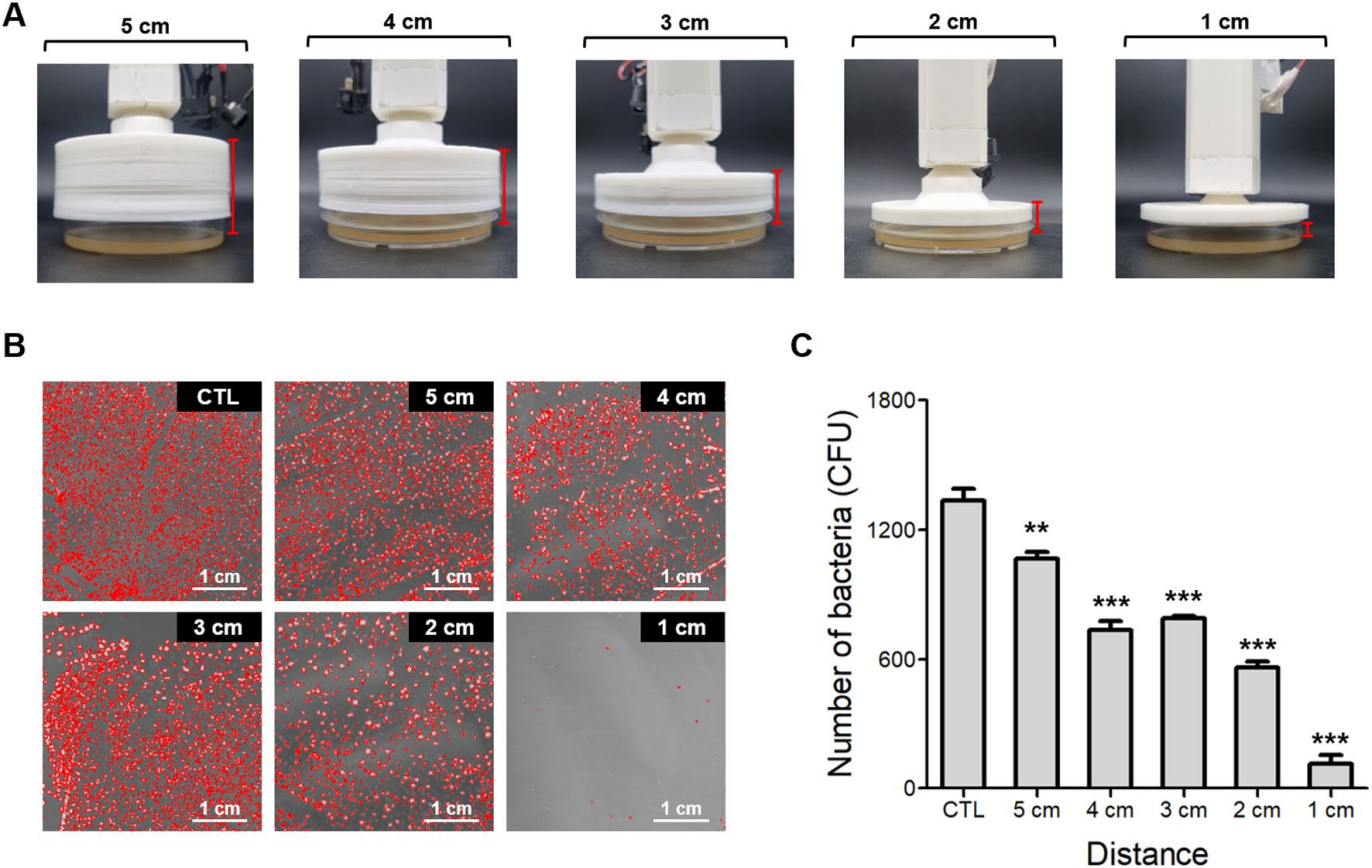
Sterilization efficiency of *Staphylococcus hominis* (*S. hominis*) based on the distance from the air emission point. (**A**) Photos of the adjustment cap for the distance between bacteria and plasma outflow. (**B**) Representative images of *S. hominis* after 1 min of plasma discharge. (**C**) Graph showing the colony counts. Error bars in the mean graphs were calculated using the same method as for the six plates. All t-test values indicate p < 0.001 compared to the control, except at 5 cm (p = 0.0047).

To evaluate the functional activity of the deodorant, we examined its antibacterial effects after plasma discharge. Figure 3 shows the antibacterial effect based on the distance of plasma discharge from *S. hominis*, a major causative bacterium of axillary odor. To maintain the distance between the plasma device and agar plate, we prepared petri dish covers with heights ranging from 1 to 5 cm (Fig. 3A). Subsequently, we compared the antibacterial activities at various distances after 1 min of plasma discharge. As shown in Fig. 3B, bacterial colonies are highlighted in red using the ImageJ program to facilitate the counting of the remaining bacteria after plasma discharge. This result shows that the quantity of remaining bacteria is contingent on the height of the plasma discharge compared to the control. Plasma exposure significantly inhibited *S. hominis* growth in a discharge distance-dependent manner (Fig. 3C). This suggests that the plasma device is effective in killing bacteria within 5 cm of the target surface.

**Figure 4:**
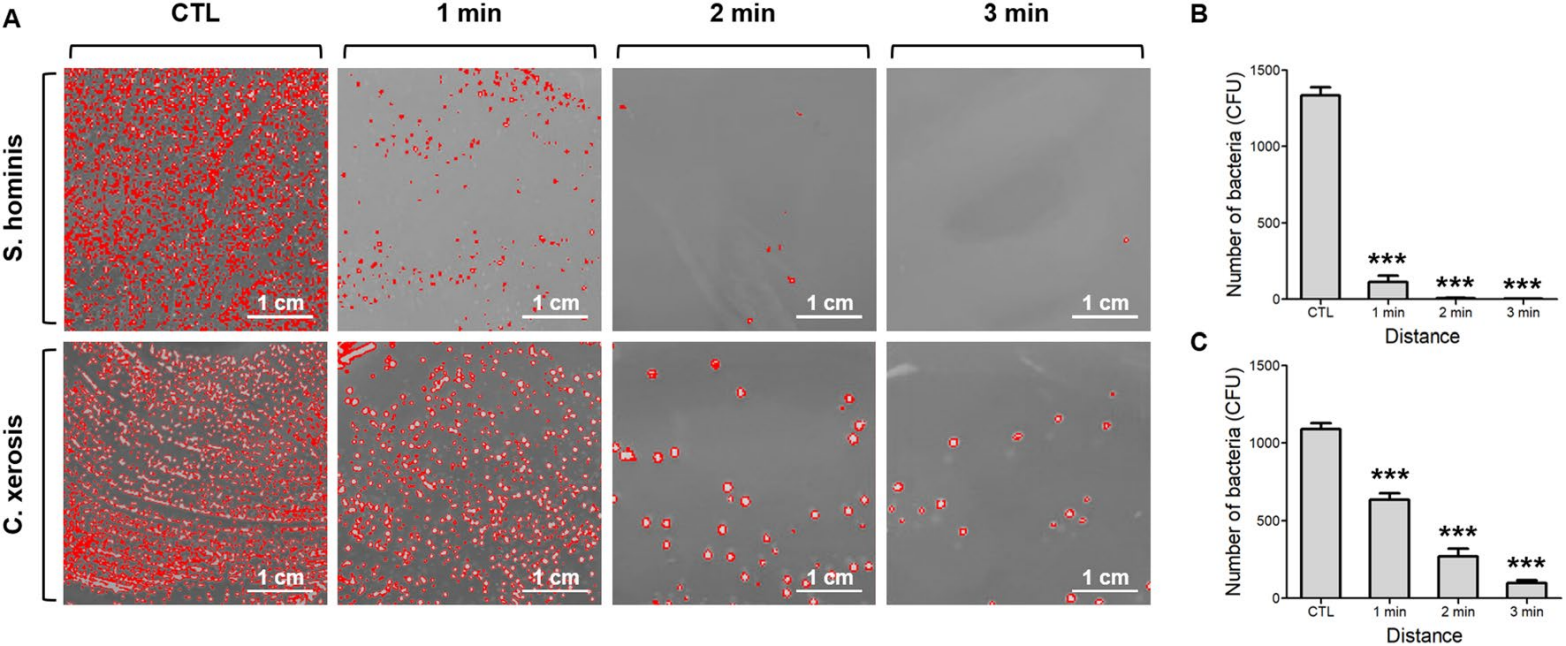
Sterilization efficiency of *S. hominis* and *C. xerosis* based on discharge time. (**A**) Representative sterilization images (**B**) Colony counts of *S. hominis* (**C**) Colony counts of *C. xerosis*. Error bars in the mean data were calculated using the same method as for the eight plates. All t-test values indicate p < 0.001 compared to the control, except at 1 min for *C. xerosis* (p = 0.0002).

In addition, the duration of the plasma discharge is a critical factor for antibacterial activity. Therefore, we further investigated the antibacterial effects against both *S. hominis* and *C. xerosis* based on varying plasma discharge durations. Figure 4A shows representative images of the remaining *S. hominis* and *C. xerosis* bacteria over exposure times of 1, 2, and 3 min at a distance of 1 cm. As shown in Fig. 4B, significant differences in the number of *S. hominis* colonies between the control and treatment groups were evident 1 min after discharge (p < 0.001). As the distance decreased, the bacterial colony count decreased significantly, reaching an inhibition percentage of 99.81% for *S. hominis* after 3 min of exposure. Similarly, the difference in the colony number of *C. xerosis* was also significant from the 1-min discharge, reaching an inhibition percentage of 90.99% after 3 min of exposure (Fig. 4C). These findings indicated an antibacterial activity of over 90% against axillary odor-producing *S. hominis* and *C. xerosis* 3 min after discharge.

**Figure 5:**
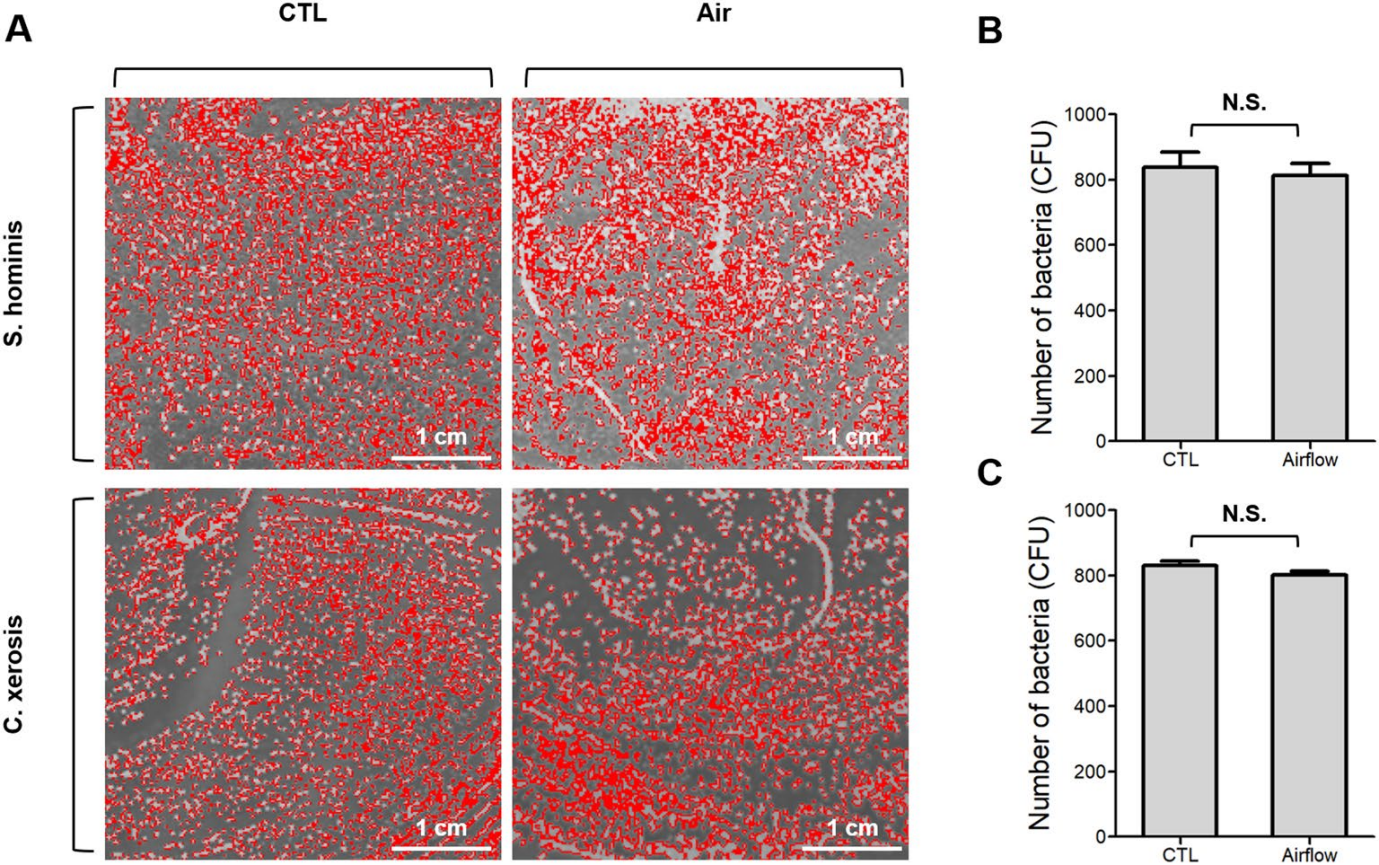
Airflow effect on sterilization of *S. hominis* and *C. xerosis*. (**A**) Representative sterilization images (**B**) Colony counts of *S. hominis*; (**C**) Colony counts of *C. xerosis*. No significant impact was observed (ns).

In our plasma device, the circulating airflow during plasma discharge can inhibit bacterial growth due to the dehydration of the agar plate as a false-positive effect. Therefore, we compared the antibacterial activity after airflow exposure without plasma discharge (Fig. 5A). There was no significant antibacterial effect against *S. hominis* (Fig. 5B) or *C. xerosis* (Fig. 5C) after exposure to airflow for 3 min at a distance of 1 cm. This supports the idea that the antibacterial effect of our deodorant can be attributed to plasma irradiation.

### Deodorizing effect of plasma deodorant

**Figure 6:**
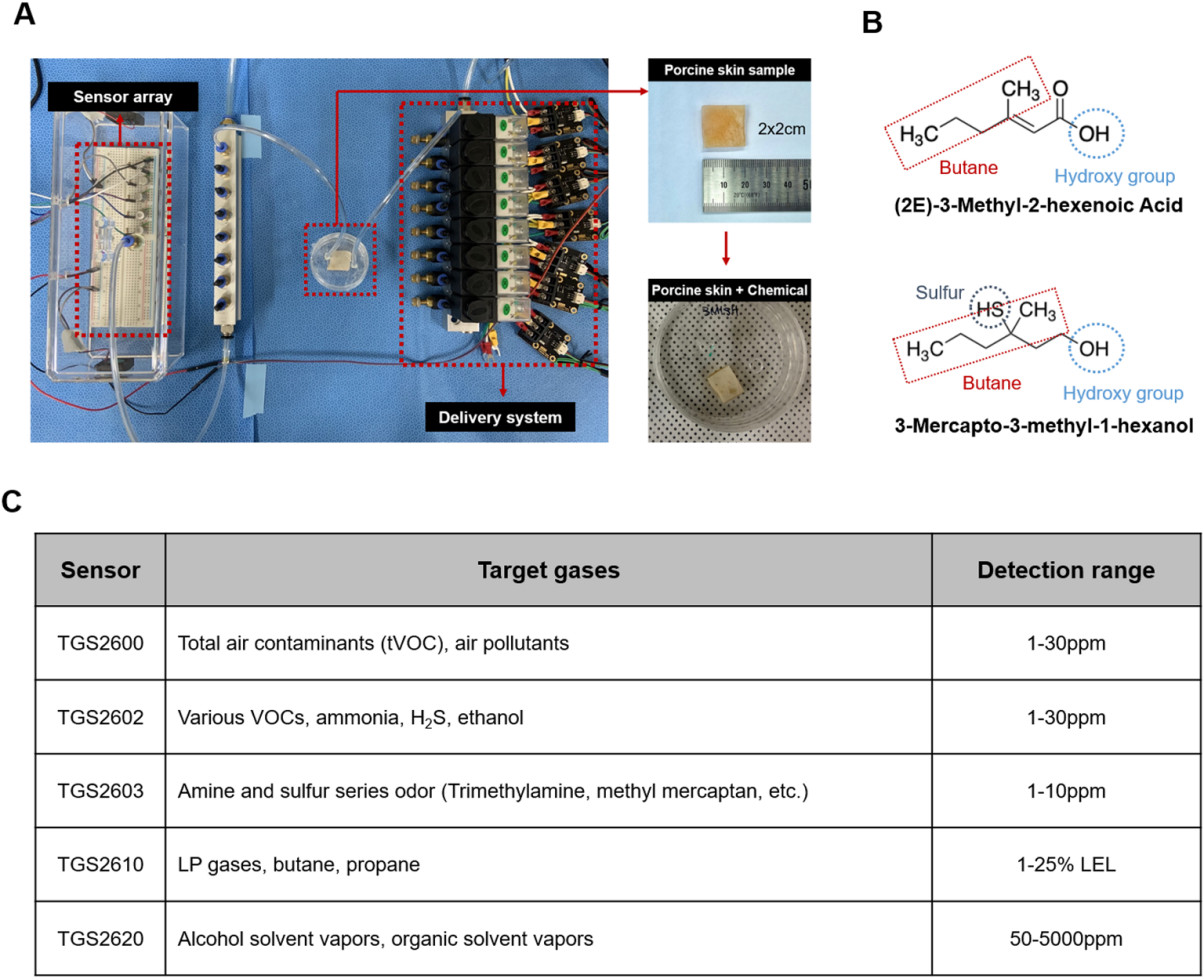
The configuration of the electronic nose system. (**A**) Image of the electronic nose system used to measure the odor of porcine skin samples (**B**) VOC compounds, HMHA, and 3M3SH used in this experiment to induce axillary odor (**C**) The sensors in the array are designed to target gases related to the measurement of axillary odor, such as VOCs, sulfur compounds, and butane.

The deodorizing effects of the deodorants were assessed using an electronic nose system. Figure 6A shows the electronic nose system, which consists of odor delivery and gas sensor array components. It is well-known that 3-hydroxy-3-methylhexanoic acid (3M2H) and 3-methyl-3-sulfanylhexan-1-ol (3M3SH) serve as representative compounds in axillary odor^24–26^. As shown in Fig. 6B, these compounds contained butane, hydroxyl, and/or sulfur groups in their chemical structures. Therefore, the sensor array consists of gas sensors sensitive to butane, hydroxyl, and/or sulfur groups (Fig. 6C). 3M2H and 3M3SH were applied to porcine skin to mimic the axillary odor and were subsequently placed within the sample chamber with inlet and outlet connections. The inlet hose connected to the air delivery component was responsible for introducing 3M2H or 3M3SH into the sample chamber, whereas 3M2H or 3M3SH was transported and measured within the sensor array chamber through the outlet hose.

**Figure 7:**
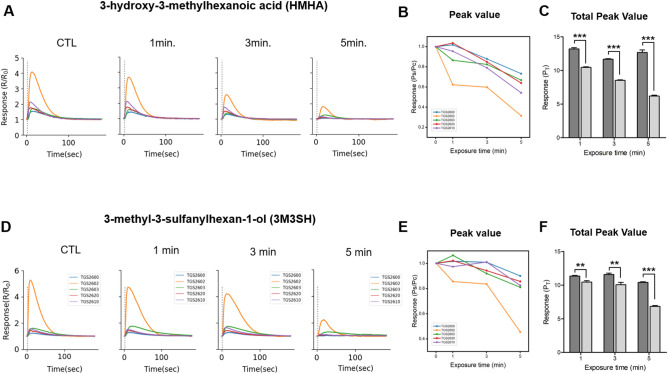
Deodorizing effect of the plasma device. (**A**) Resistance peak graphs of each sensor with respect to plasma discharge time for HMHA; (**B**) Reduction ratio (Ps/Pc) of peak values for HMHA compared to the control; (**C**) Total peak value (PT) for HMHA compared to the control. All t-test values indicate p < 0.001 compared to the control; (**D**) Resistance peak graphs of each sensor with respect to plasma discharge time for 3M3SH; (**E**) Reduction ratio (Ps/Pc) of peak values for 3M3SH compared to the control; (**F**) Total peak value (PT) for 3M3SH compared to the control. All t-test values indicate p < 0.001 compared to the control, except at 1 min and 3 min for 3M3SH (p = 0.0025 and 0.0027, respectively).

To evaluate the deodorizing effect, we compared the sensor array readings after plasma exposure for 1, 3, and 5 min with those of the control group. The control group was exposed to the same airflow exposure time without plasma irradiation. Figure 7A,D show the responses to 3M2H and 3M3SH after plasma exposure for 1, 3, and 5 min, respectively. The peak values of 3M2H (Fig. 7B) and 3M3SH (Fig. 7E) in response to each sensor decreased in an exposure-time-dependent manner. When compared with the total response of all sensors, the odoriferous 3M2H (Fig. 7C) and 3M3SH (Fig. 7F) were significantly reduced at all time points compared with those of the control group. Notably, both 3M2H and 3M3SH were removed by approximately 70% and 73% after a 5 min plasma exposure, respectively.

## Conclusions

This study introduces a novel electronic deodorant with a new concept, applying cold atmospheric plasma technology to treat axillary odor. Cold Plasma discharge temporarily generated neutral particles, ions, radicals, electrons, and ultraviolet (UV) radiation^8,9^. Due to its high nitrogen-to-oxygen ratio at atmospheric pressure, it generates ROS and RNS. These active species have known to disrupt the outer membrane of the bacteria, resulting in sterilization. Other reactive species, including ozone, had been found to transform odorous volatile organic compounds (VOCs) into alternative degradation products, resulting in efficient VOC removal. Therefore, non-thermal plasma technology presented a remarkable potential to address both bacterial sterilization and odor elimination through a non-contact approach without the need for chemical agents^8,9,12–17^. In cases of axillary bromhidrosis, where both these functions are required, cold atmospheric plasma technology can be applied to deodorant devices for treatment and management. The primary advantageous aspect of cold plasma is its safety, as the active species it generates are promptly degraded or neutralized. Nevertheless, it is internationally regulated through standards that govern ozone generation in plasma devices to mitigate potential health risks. When the distance from the armpit to the nose was approximately 30 cm, the ozone concentration measured 0.16 ppm when measured in a straight line at a distance of 30 cm (Fig. 2D) and 0 ppm when measured diagonally at the same distance (Fig. 2E). These values are notably below international safety standards.

## Materials and methods

### Plasma equipment validation

The AC voltage and discharge current were measured using an oscilloscope (DSOX2014A, Keysight, USA), high-voltage probe (P6015A, Tektronix, USA), and current probe (P6022, Tektronix, USA) to compare the electrical properties of the cold atmospheric plasma.

### Plasma discharge and airflow conditions

Caps designed to maintain a consistent distance between the plasma equipment and the petri dish were tailored to fit 100 × 20 petri dishes. The caps were crafted using a 3D printer (Creator 3 Pro, FlashForge). Plasma discharge was performed at room temperature and atmospheric pressure. During the airflow experiments, the plasma discharge switch on the device was switched off. The discharge duration and type of cap used were adjusted based on the specific experimental conditions.

### Detection of plasma emission components

Plasma discharge was conducted in ambient air, and to ensure accuracy, both the discharge and measurements were conducted within a chamber. Optical emission spectra (OES) were obtained using a spectroscopy system (HR4D1252, Ocean Optics). The optic fiber was connected to the OES spectral analyzer and positioned 5 mm from the plasma device nozzle. Ozone concentration was measured using a gas detector (X-am 5000, Dräger, Germany). The deodorant device was deactivated 5 min after the operation. Measurements were taken at distances of 3, 30, and 30 cm (closed armpit) from the plasma–air outflow area.

### Heat generation temperature measurement

Frozen 5 × 6 cm pieces of porcine skin were allowed to equilibrate at room temperature for one hour. The plasma was then discharged for 5 min using a 1 cm cap. Thermal imaging of the porcine skin was performed using a FLIR E96 camera from before plasma discharge to 5 min afterward. The extent of heat generated by the plasma was determined based on the highest temperature observed in the porcine skin.

### Preparation and culturing of bacteria

This study utilized strains of *Staphylococcus hominis* (*S. hominis*) and *Corynebacterium xerosis* (*C. xerosis*) obtained from the Korean Collection for Type Cultures (KCTC) and stored at − 80 °C in a medium containing 20% glycerol. *S. hominis* was diluted in Nutrient Broth No. 1 to achieve the desired colony-forming units, and 20 µL was divided into 2 µL drops for spreading on Nutrient Broth No. 1 agar. After spreading, the appropriate cap was placed on a lidless dish, and the plasma was discharged for a specific duration. Following plasma discharge, the petri dishes were sealed and incubated at 37 °C for 24 h. *C. xerosis* was cultured in Nutrient Broth No. 2 agar using the same method. The quantity of Nutrient Broth No. 1 and Nutrient Broth No. 2 agar used in each petri dish was 20 mL and remained consistent.

### Deodorization efficacy validation

The electronic nose is composed of a control unit, sensor unit, and gas supply unit. The sensor unit consisted of five gas sensors (TGS2600, TGS2602, TGS2603, TGS2620, and TGS2610) from Figaro, USA, Inc., which were preheated for 12 h before the measurements to ensure a stable response. The odorous compounds HMHA and 3M3SH (Toronto Research Chemicals, Canada) were diluted with methanol. HMHA was used at a final concentration of 0.1 M, whereas 3M3SH was used at a final concentration of 1 M. Each diluted compound was absorbed into 2 × 2 cm pieces of porcine skin. Subsequently, the plasma was discharged for 1, 3, and 5 min from a distance of 1 cm. The control group was exposed to airflow without plasma discharge. Porcine skin was then placed in a 30 mm sealed petri dish to collect the gas sample. Ambient air was injected into the sample dish at 41.7 ml/s for 2 s, allowing delivery of the headspace sample into the sensor unit. Data were measured every 2 s with a 2 s target gas exposure and a 3 min recovery period.

## Data Availability

The datasets used and/or analyzed during the current study available from the corresponding author on reasonable request.
